# A Novel Nomogram for Predicting the Risk of Premature Delivery Based on the Thyroid Function in Pregnant Women

**DOI:** 10.3389/fendo.2021.793650

**Published:** 2022-01-10

**Authors:** Yu Meng, Jing Lin, Jianxia Fan

**Affiliations:** Department of Obstetrics and Gynecology, International Peace Maternity and Child Health Hospital, School of Medicine, Shanghai Jiaotong University, Shanghai, China

**Keywords:** thyroperoxidase antibody, thyroid-stimulating hormone, premature delivery, nomogram, pregnancy trimester, first

## Abstract

**Background:**

Maternal thyroid dysfunction and autoantibodies were associated with preterm delivery. However, recommendations for cutoff values of thyroperoxidase antibody (TPOAb) positivity and thyroid-stimulating homone (TSH) associated with premature delivery are lacking.

**Objective:**

To identify the pregnancy-specific cutoff values for TPOAb positivity and TSH associated with preterm delivery. To develop a nomogram for the risk prediction of premature delivery based on maternal thyroid function in singleton pregnant women without pre-pregnancy complications.

**Methods:**

This study included data from the International Peace Maternity and Child Care Health Hospital (IPMCH) in Shanghai, China, between January 2013 and December 2016. Added data between September 2019 and November 2019 as the test cohort. Youden’s index calculated the pregnancy-specific cutoff values for TPOAb positivity and TSH concentration. Univariate and multivariable logistic regression analysis were used to screen the risk factors of premature delivery. The nomogram was developed according to the regression coefficient of relevant variables. Discrimination and calibration of the model were assessed using the C-index, Hosmer-Lemeshow test, calibration curve and decision curve analysis.

**Results:**

45,467 pregnant women were divided into the training and validation cohorts according to the ratio of 7: 3. The testing cohort included 727 participants. The pregnancy-specific cutoff values associated with the risk of premature delivery during the first trimester were 5.14 IU/mL for TPOAb positivity and 1.33 mU/L for TSH concentration. Multivariable logistic regression analysis showed that maternal age, history of premature delivery, elevated TSH concentration and TPOAb positivity in the early pregnancy, preeclampsia and gestational diabetes mellitus were risk factors of premature delivery. The C-index was 0.62 of the nomogram. Hosmer-Lemeshow test showed that the Chi-square value was 2.64 (*P* = 0.955 > 0.05). Decision curve analysis showed a positive net benefit. The calibration curves of three cohorts were shown to be in good agreement.

**Conclusions:**

We identified the pregnancy-specific cutoff values for TPOAb positivity and TSH concentration associated with preterm delivery in singleton pregnant women without pre-pregnancy complications. We developed a nomogram to predict the occurrence of premature delivery based on thyroid function and other risk factors as a clinical decision-making tool.

## Introduction

Premature delivery is defined as delivery before gestation week 37 (1). Every year, there are 15 million preterm births worldwide, responsible yearly for 965,000 neonatal and 125,000 toddlers and preschool children (aged 1 – 5 years) deaths (1, 2). The frequency of premature births is reported as 12 ~ 13% in America and 5 ~ 9% in the other developed countries (3). The causes of premature delivery are mostly unclear, and our knowledge of its pathophysiology is still limited. Despite the socio-demographic, environmental, obstetric, fetal and medical factors were reported to be associated with premature births, approximately two-thirds of premature births occur without an obvious risk factor (4–8).

Clinicians need a simple algorithm to identify pregnant women at the risk of premature delivery by applying it to all symptomatic or asymptomatic patients at any given gestational age, those with a singleton at high risk, and those at low risk (9). Unfortunately, it is unlikely that a single test could predict all premature deliveries. Recently, clinical risk prediction models are developed to predict the probability of preterm delivery in pre-pregnancy women or high risk populations (10–13). However, singleton pregnant women without any pre-pregnancy complications, as a low risk group, lack an individualized assessment or prediction model for the risk of premature birth.

Dysfunction of maternal thyroid is relatively common during pregnancy (14). Overt hyperthyroidism and hypothyroidism are well-known risk factors for premature delivery (15). Thyroid autoimmunity (TAI) is much more frequent in pregnant women than overt thyroid diseases, with a prevalence of 10% for thyroperoxidase antibody (TPOAb) positivity (16). Recent Studies showed that TPOAb-positive pregnant women had a significantly high risk of premature delivery (14). The pathophysiological mechanisms underlying this association are still unknown but are suspected to include subtly impaired thyroid function, a direct effect of thyroid autoantibodies on fetal tissue or an underlying more generalized autoimmune dysfunctions (17). Thyroid autoantibodies can reflect a generalized activation of the immune system and specifically a dysregulated activity of the immune system at the fetal-maternal interface (18). Dysregulation of the local placental-decidual environment can be associated with miscarriage and premature delivery (19). Thyroid function screening during pregnancy should include at least an assessment of thyroid-stimulating hormone (TSH) and TPOAb concentrations, regardless of the screening method (20). We hypothesized that TPOAb positivity and TSH concentration could be novel markers of premature delivery in pregnant women.

Taking into account the unique changes of maternal thyroid function in the first half of pregnancy, the latest American Thyroid Association (ATA) guidelines advocated to use pregnancy-specific and regional reference ranges for free thyroxine (FT4) and TSH based on euthyroid pregnant women (21). But the cutoff value for TPOAb positivity is usually provided by the assay manufacturer. However, it is unknown whether such a cutoff value could be generalized to the pregnant population. The determination of TPOAb positive cutoff value in previous studies did not fully consider the thyroid function changes in pregnancy, and usually has a wide threshold range (22). There are no data on reference ranges for pregnancy-specific TPOAb and TSH concentration in association with premature delivery.

This study aimed to determine the pregnancy-specific cutoff values for TPOAb-positive and TSH concentration association with the risk of premature delivery in singleton pregnant women without pre-pregnancy complications. Furthermore, we aimed to construct a nomogram to predict premature delivery based on thyroid function and other risk factors. The aim of our study was to develop a clinical decision-making tool for assessing the individual risk for premature delivery in pregnant women.

## Materials and Methods

### Patient Enrollment

The retrospective study was performed at the International Peace Maternity and Child Health Hospital (IPMCH), a large public hospital providing tertiary care in Shanghai, China. The project was approved by the Ethics Committee of IPMCH (No. GKLW2019–16). From January 1, 2013, to December 31, 2016, a total of 52,027 pregnant women were enrolled the cohort. We added data from the same institution between September 2019 and November 2019 as the test cohort. Women who met the following criterias were included: participants who underwent a first prenatal screening during the first trimester at IPMCH and their FT4, TSH, and TPOAb data from the first presentation were available. Women with chronic diseases are known to cause adverse pregnancy outcomes and interventions are expected to be required before conception and during pregnancy. The exclusion criteria were as follows (1): women who had a history of thyroid diseases, diabetes mellitus, chronic hypertension before pregnancy; (2) those using medication known to interfere with thyroid function before or after baseline measurements; (3) pregnant women with miscarriages or multiple births, induced abortions, or stillbirths, as the gestational age or birth weight were unavailable for these neonates. As a result, 45,467 pregnant women were enrolled in this study. Then randomly divide all the enrolled participants into the training cohort (n = 31,827) and validation cohort (n = 13,640) according to the ratio of 7: 3. 727 pregnant women were enrolled as the test cohort. The regional iodine status of pregnant women in Shanghai is considered adequate during the first trimester [urinary iodine concentration (UIC), 155.0 μg/L] and second trimester (UIC, 151.0 μg/L) (23).

### Data Collection

The data came from the electronic medical record system of IPMCH. Data on maternal age, parity, last menstrual period (LMP), education levels, and previous diseases such as chronic hypertension, diabetes mellitus were collected through the first interview (about 9 – 13 weeks of pregnancy). Gestational age determination was estimated based on the date of LMP and confirmed by ultrasound. Fasting blood samples were drawn from the median cubital vein, and the serum was separated by centrifugation within six hours. The measurements of FT4, TSH and TPOAb concentrations were obtained in early pregnancy (9 – 13 weeks) and measured using the Architect i2000 immunoassay (Abbott, Chicago, IL, USA) according to the manufacturer’s protocols. The intra- and inter-assay coefficient of variation ranged between 1.6 – 3.6% for TSH, 1.9 – 4.0% for FT4, and was 10.0% for TPOAb positivity (24). Information on pregnancy outcomes such as gestational age, birth weights and pregnancy complications was also obtained from the electronic medical records.

### Diagnostic Criteria

Maternal pre-pregnancy body mass index (BMI) was calculated by dividing the pre-pregnancy weight (kg) by the squared height (m^2^). Gestational hypertension was defined as new-onset hypertension without proteinuria, with blood pressure (BP) of ≥140/90 mmHg after week 20 of gestation; preeclampsia was defined by the same criteria and with proteinuria of 1+ on dipstick testing occurring when the BP was elevated (25). Gestational diabetes mellitus (GDM) was conducted with an abnormal oral glucose tolerance test (OGTT) at week 24 – 28 of gestation and defined following the standard diagnostic criterias that established by the American Diabetes Association (26). The main pregnancy outcome was the gestational age of the neonates. Premature delivery was defined as delivery before gestational week 37 or with a birth weight more than 1,000 g.

### Statistical Analysis

The data were analyzed using IBM SPSS Statistics for Windows, Version 25.0 (IBM Corp., Armonk, NY, USA) and R 4.1.1. The measurement data were shown as mean ± standard deviation (SD) and statistically compared by the independent samples *t*-test. The categorical data were expressed as count and percentage and compared by the chi-squared test. The relationships between the risk factors and premature delivery were analyzed using the univariate and multivariable logistic regression analysis. Each independent variable was examined by a univariate model. Variables associated with the studied outcome (*P* < 0.05) would be included in the multivariate model. We used the maximum Youden’s index for rating diagnostic tests to calculate the optimal cut-off point of TPOAb positivity and TSH concentration related to the prevalence of premature delivery. Then randomly divide all the enrolled participants into the training cohort (n = 31,827) and validation cohort (n = 13,640) according to the ratio of 7: 3. The test cohort included 727 pregnant women. The nomogram was developed based on the regression coefficients of the relevant variables in the training cohort. The values for model covariates were mapped to points in the range of 0 to 100. The total number of points obtained by the predictive model corresponded to the prevalence of premature delivery. Discrimination and calibration of the model were assessed using the C-index, Hosmer-Lemeshow test, calibration curve and decision curve analysis. Decision curve analysis was used to determine the clinical utility of the prediction model. The decision curve plots net benefits for a range of relevant risk thresholds. The performance of the nomogram was evaluated by the calibration curve in the validation cohort and test cohort. The closer the dots were to the diagonal dotted line, the better the prediction model was. Pregnancy outcomes included gestational week, birth weight of newborns, and premature delivery rate. The logistical regression model was used to estimate the odds ratios (ORs), hazard ratios (HRs) and 95% confidence intervals (CI) for the association between variables and preterm delivery. P value < 0.05 was considered statistically significant.

## Results

### Patient Characteristics

**Figure 1:**
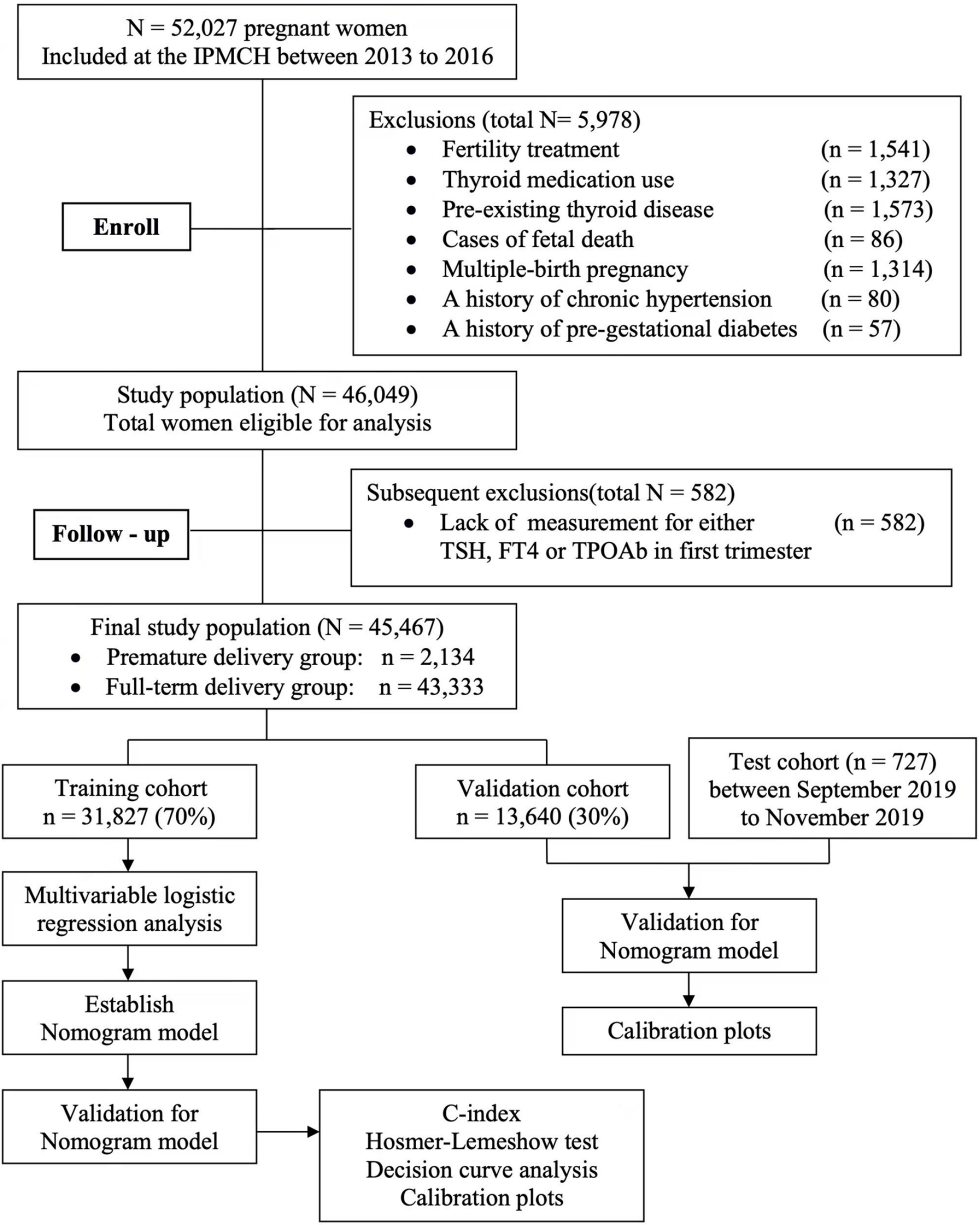
Flowcharts illustrating study population selection and data availability.

**Figure 1** showed the flow diagram for inclusion and exclusion of the study. Finally, 45,467 eligible pregnant women were enrolled in this study. Premature delivery occurred in 2,134 women (4.7%). The pregnant women were divided into two groups according to the gestational week of neonates. The patient characteristics in both groups are shown in **Table 1**. The minimum maternal age was 18 years and the maximum maternal age was 49 years. The mean maternal age was 30.68± 3.82 years in premature-delivery group and 30.01 ± 3.56 years in full-term group (*P* < 0.001). The median pre-pregnancy BMI was 21.18 ± 2.98 kg/m^2^ in premature-delivery group and 20.99 ± 2.72 kg/m^2^ in full-term group (*P* = 0.023). The proportion of multiparous women was higher in the premature delivery group (21.0% vs. 18.3%, *P* = 0.001). The proportion of previous history of premature delivery was higher in the premature-delivery group (1.7% vs. 0.3%, *P* < 0.001). Maternal education level and smoking were similar between two groups.

**Table 1:** Patient baseline characteristics.

Characteristics	Full-term delivery	Premature delivery	*P*-value
Number (%)	43,333 (95.3)	2,134 (4.7)	
Age^a^ (years)	30.01 ± 3.56	30.68 ± 3.82	< 0.001
Pre-pregnancy BMI^a^ (kg/m^2^)	20.99 ± 2.72	21.18 ± 2.98	0.023
Parity^b^ (n, %)			0.001
Primiparous	35,406 (81.7)	1,685 (79.0)	
Multiparous	7,927 (18.3)	449 (21.0)	
Smoking^b^ (n, %)			0.651
No	43,301 (99.9)	2,133 (100.0)	
Yes	32 (0.1)	1 (0.0)	
Education level^b^ (n, %)			0.063
Bachelor degree and below	35,331 (81.5)	1,774 (83.1)	
Master degree and above	8,002 (18.5)	360 (16.9)	
History of premature delivery^b^ (n, %)			< 0.001
No	43,212 (99.7)	2,097 (98.3)	
Yes	121 (0.3)	37 (1.7)	

a Mean ± SD, compared by independent-samples t-test.

b Compared by the chi-squared test.

BMI, body mass index.

### Assessment of the Pregnancy-Specific Cutoff Values for TPOAb Positivity and TSH Concentration Associated With Premature Delivery

The Youden’s index was used to calculate the pregnancy-specific and regional cutoff values for TPOAb positivity and TSH concentration related to the prevalence of premature delivery. The cutoff value for TPOAb positive in the first trimester based on Youden’s index maximum was 5.14 IU/mL, lower than the manufacturer’s cutoff (5.61 IU/mL). The cutoff value for TSH concentration in the first trimester based on Youden’s index maximum was 1.33 mU/L, significantly lower than the upper limit of normal threshold value for TSH in euthyroid pregnant women (3.52 mU/L).

### Logistic Regression Analysis and Development of a Nomogram Prediction Model

**Figure 2:**
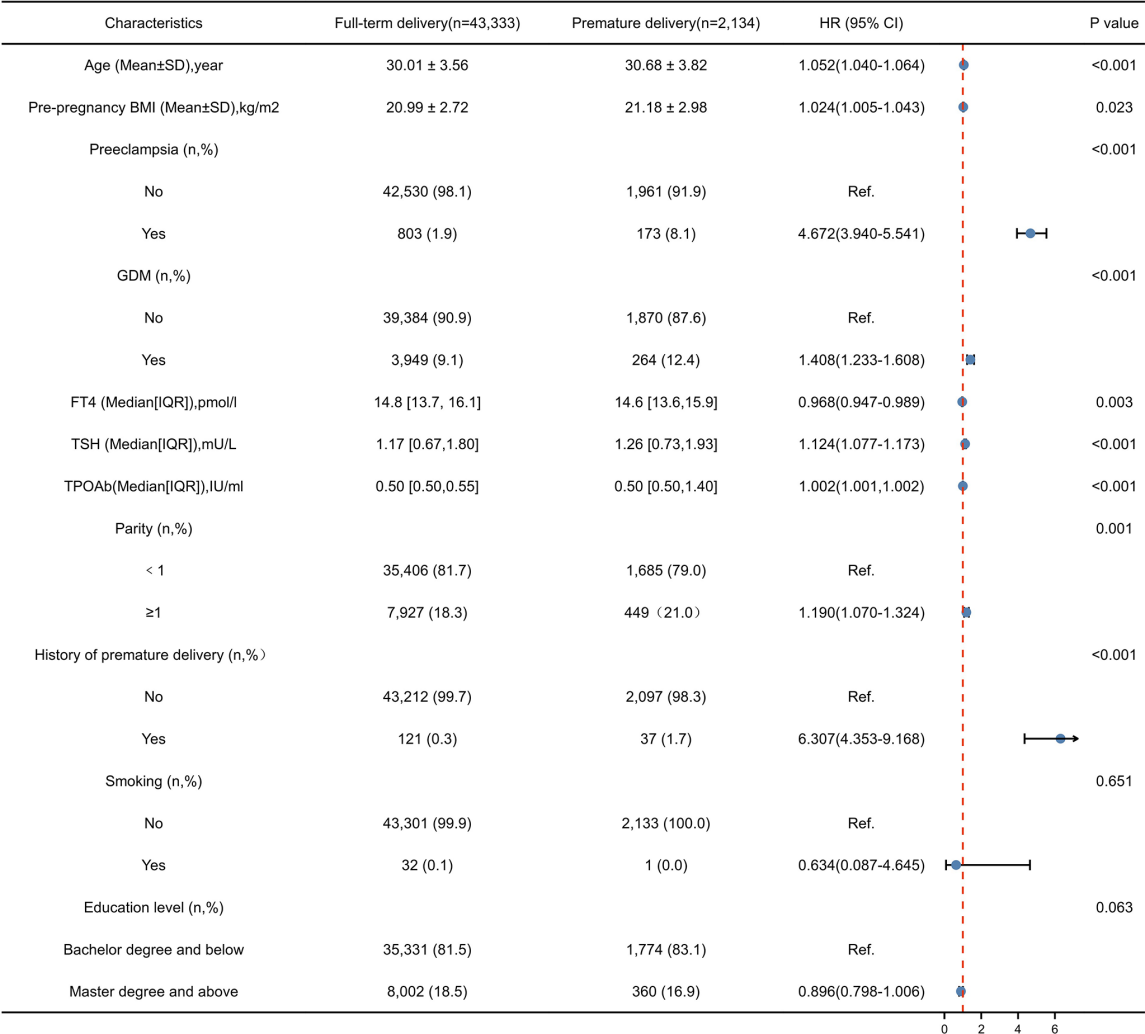
Univariate analysis of risk factors for premature delivery.

**Figure 3:**
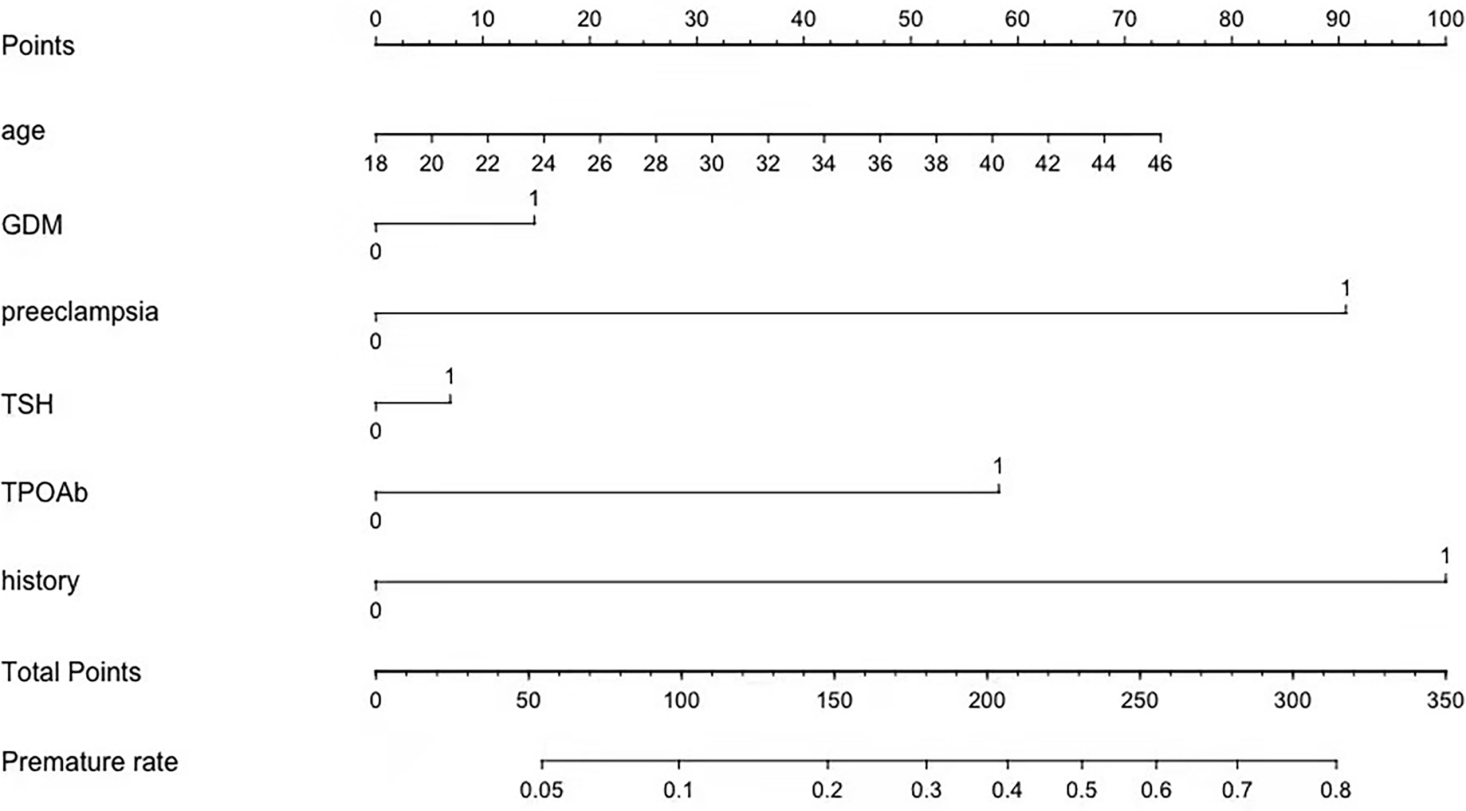
A profile of a nomogram to estimate the risk of premature delivery in the training cohort. To calculate the probability of preterm delivery, draw a line perpendicular to the corresponding axis of each risk factor until it reaches the top line labeled “Points.” Sum up the number of points for all risk factors, and then draw a line descending from the axis labeled “Total Points” until it intercepts with the lower line where the preterm birth probability is indicated. GDM, gestational diabetes mellitus; TSH, thyroid-stimulating hormone; TPOAb,thyroid peroxidase antibody; History, history of premature delivery. For Age, number=years. For binary variables, 0 = no and 1 = yes. For TPOAb positivity, 0= when TPOAb concentration less than 5.14 IU/mL and 1=when TPOAb concentration more than 5.14 IU/mL. For TSH concentration, 0= when TSH concentration less than 1.33 mU/L and 1= when TSH concentration more than 1.33 mU/L.

As shown in **Figure 2**, the univariate analysis showed that there were significant differences in maternal age, parity, history of preterm birth, pre-pregnancy BMI, preeclampsia, GDM, and TSH, FT4, and TPOAb concentrations in the first trimester between two groups. The multivariable logistic regression analysis demonstrated that maternal age, previous history of premature delivery, preeclampsia, GDM and TSH and TPOAb concentrations in the first trimester were independent risk factors for premature delivery in the training cohort (**Table 2**). The prediction model was developed based on these factors and presented as a nomogram (**Figure 3**).

**Table 2:** Multivariable analysis of risk factors for premature delivery in the training group.

Characteristic	B	SE	Wald	P	OR	95%CI
Maternal age, year	0.022	0.006	13.447	< 0.001*	1.022	1.010 − 1.034
Pre-pregnancy BMI, kg/m^2^	0.017	0.012	2.129	0.145	1.017	0.994 − 1.040
Preeclampisa	1.176	0.088	179.190	< 0.001*	3.241	2.728 − 3.850
GDM	0.279	0.093	8.971	0.003*	1.321	1.101 − 1.585
FT4^a^, pmol/L	−0.001	0.017	0.002	0.996	0.999	0.966 − 1.034
TSH^a^, mU/L	0.127	0.046	7.707	0.006*	1.135	1.038 − 1.242
TPOAb^a^, IU/mL	0.576	0.055	15.900	< 0.001*	1.001	1.001 − 1.002
History of premature delivery	1.425	0.192	54.924	< 0.001*	4.157	2.852 − 6.060

*Significant variables.

a the value was measured in the first trimester.

BMI, body mass index; GDM, gestational diabetes mellitus; FT4, free thyroxine; TSH, thyroid-stimulating hormone; TPOAb, thyroid peroxidase antibody; OR, odds ratio; CI, confidence Interval; SE, standard error.

### Apparent Performance and Clincial Use of the Nomogram

**Figure 4:**
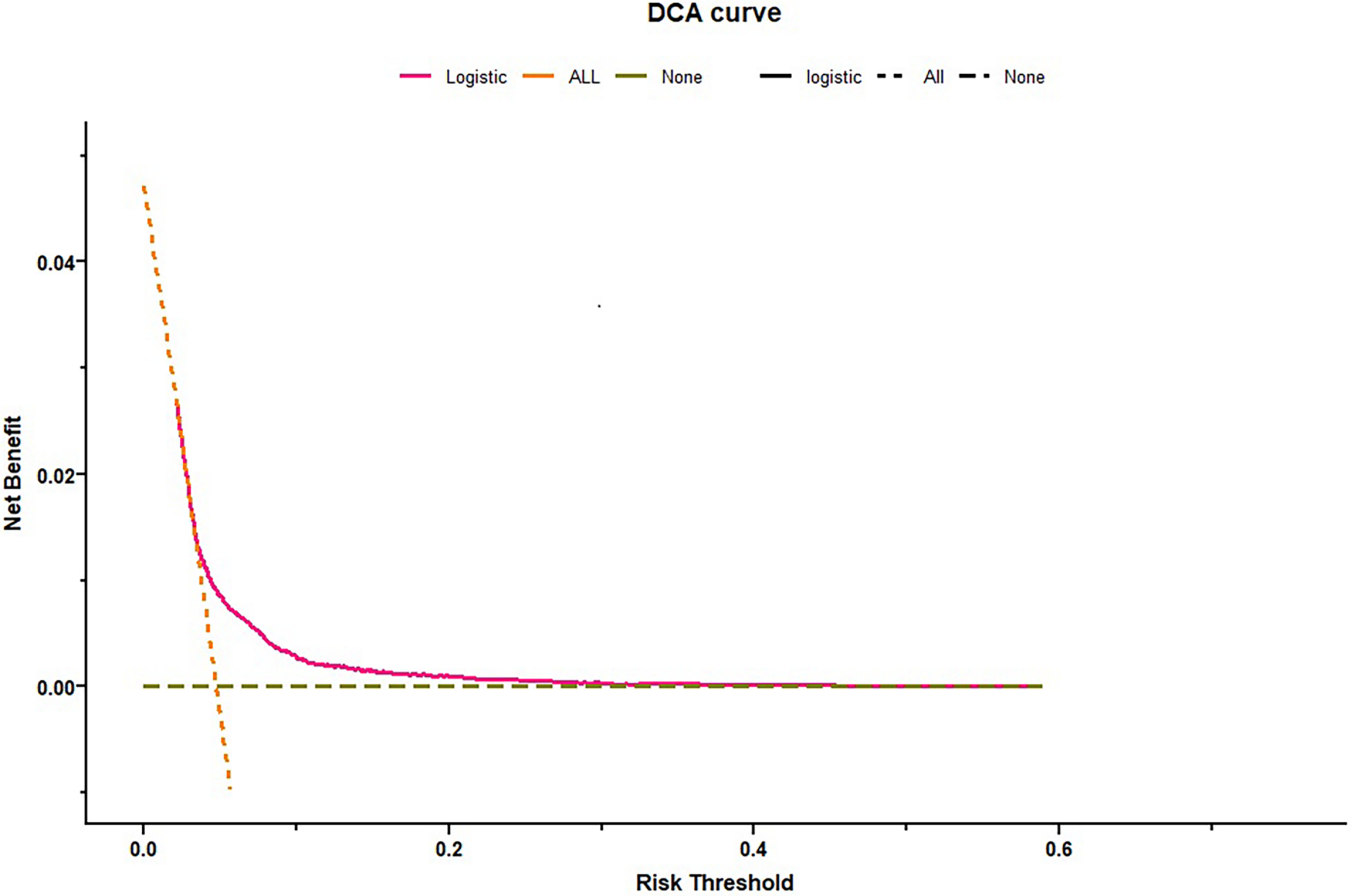
Decision curve analysis for premature delivery. Dotted green line = net benefit when no one is at risk for premature delivery; Dotted orange line = net benefit when all are at risk for premature delivery. The y-axis measures the net benefit. The red line represents the nomogram. The decision curve showed that if the threshold probability is between 0.05–0.40, using the nomogram in the current study to predict premature delivery adds more benefit than the intervention-all-patients scheme or the intervention-none scheme.

**Figure 5:**
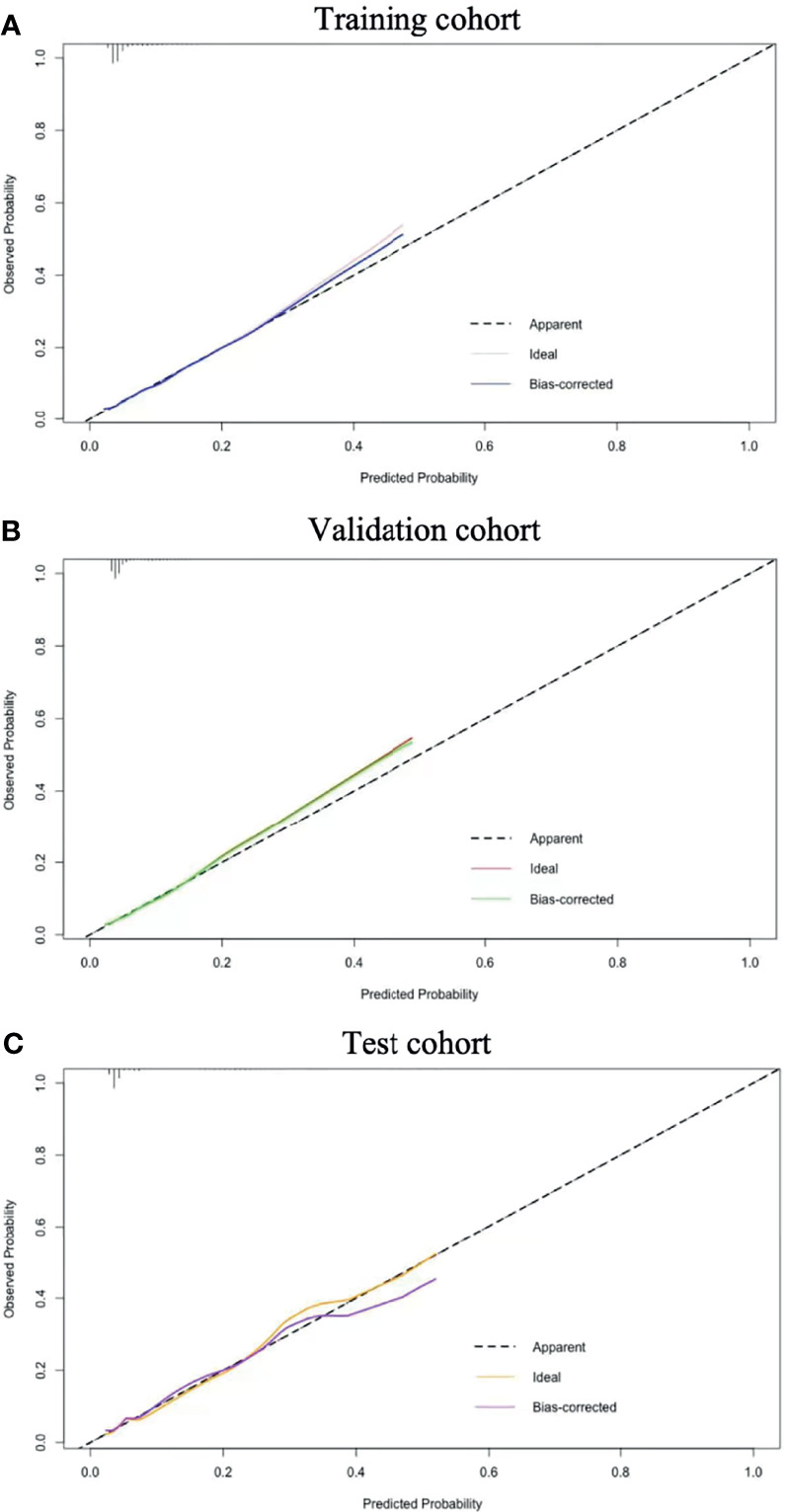
Calibration results. Nomogram-predicted probability of premature delivery is plotted on the x-axis; actual probability of premature delivery is plotted on the y-axis. The diagonal dotted line represents a perfect prediction by an ideal model. The solid line represents the performance of the nomogram. The closer this line is to the diagonal dotted line, the better the prediction. Training cohort **(A)**; Validation cohort **(B)** and Test cohort **(C)**.

The C-index was 0.62 and Hosmer-Lemeshow test for evaluation of calibration showed that the Chi-square value was 2.64 (*P* = 0.955 > 0.05) of the predictive model. Decision curve analysis indicated the net benefit of the nomogram was higher with the probability threshold ranging from 5% to 40% (**Figure 4**). The calibration curve of the training cohort was shown in **Figure 5A** (Mean absolute error = 0.002, Quantile of absolute error = 0.003). The calibration curve of the validation cohort was shown in **Figure 5B** (Mean absolute error = 0.002, Quantile of absolute error = 0.004) and the test cohort was shown in **Figure 5C** (Mean absolute error = 0.003, Quantile of absolute error = 0.007). The calibration curve of the nomogram for the prediction of premature delivery risk were proven to be in good agreement.

## Discussion

During recent decades, many studies on thyroid dysfunction and TAI in pregnant women have been published. A meta-analysis on TPOAb-positive pregnant women from 2010 (27) and a systematic review with 3,043 TPOAb-positive cases from 2019 (14) indicated a 1.69-fold and 1.33-fold higher risk of preterm delivery, respectively, in comparison to TPOAb-negative pregnant women. These studies have demonstrated the importance of the underlying pathophysiological mechanisms. Compared with the non-pregnant population, TSH measurement declines in the early pregnancy and gradually increases throughout the later pregnancy, peaking just before delivery (28). TAI causes a gradual decrease in thyroid functional capacity and the adverse effects on thyroid function may be begun in the first trimester (20). TPOAb and its production might be a response to thyroid injury rather than a cause of it. The slightly abnormalities of maternal thyroid function might be related to the dysfunction of maternal-placental unit (29). The maternal-placental unit provides a stringently regulated endocrine and metabolic network for the fetus (30). Dysfunction of the maternal thyroid seems to be associated with an impairment of the placental-fetal glucose metabolism that might predispose the fetus to hypoglycemia and growth retardation by increasing the risk of low birth weight (31). The pathophysiological mechanism between maternal TAI and premature delivery needs further research.

Whether the metabolic control was achieved before and during pregnancy resulted in different pregnancy outcomes. However, studies on thyroid dysfunction and premature delivery have yielded mixed results. The exclusion or inclusion criterias used to select the pregnant women with thyroid disorders might be the major causes of heterogeneity in these studies (27). The 2017-ATA guidelines advocate using pregnancy-specific and regional reference ranges for FT4 and TSH measurements, but the definition of the regional and pregnancy-specific reference range for TPOAb positivity was not mentioned (20). Studies have reported various upper limits for TSH measurement (ranging from >2.5 to >6.0 mU/L) (14) and a wide range of cutoff values (ranging from 15 to 143 IU/mL) are used to define TPOAb positivity (22). The differences between assays and cutoffs used in various studies could contribute to the differences in the reported prevalence of thyroid antibodies, and differences in antibody associations with pregnancy outcomes (17). It was found that a dose-dependent relationship between TPOAb and thyroid function as well as the risk of premature delivery (22). In previous study, we have established a regional and pregnancy-specific thyroid function reference ranges for euthyroid pregnant women, 3.52 mU/L as an upper limit threshold value for TSH measurement in the first trimester (32). However, the regional pregnancy-specific cutoff value for TPOAb positivity has not been established yet. In our study, we investigated the association between maternal thyroid dysfunction and premature delivery. First, our study showed TPOAb-positive and TSH concentration during the first trimester was significantly associated with premature delivery. Furthermore, we investigated pregnancy-specific and regional cutoff values for TPOAb positivity and TSH concentration during the first trimester associated with premature delivery. The cutoff value for TPOAb positivity was lower than that provided by the assay manufacturer for the normal non-pregnant population (5.14 vs. 5.61 IU/mL). The cutoff value for TSH concentration was considerably lower than the upper limit of threshold value for TSH concentration in euthyroid pregnant women (1.33 vs. 3.52 mU/L). The risks indicated by these persisted even after adjusting for other confounding factors. Therefore, our study indicated that TPOAb positivity and TSH concentration in the early pengnancy were independent risk factors for premature delivery.

Our nomogram not only included the major well-known risk factors of preterm delivery, but also included other newly identified risk factors such as thyroid function, that had not been used in a nomogram associated with premature delivery before. Maternal history of premature delivery was commonly reported to confer a higher risk of preterm delivery in subsequent pregnancies (3, 8). Laughon et al. showed that previous history of premature birth was the most important risk factor for premature delivery, associated with a 32% high risk of a recurrent preterm birth (33). Previous preterm delivery in this study was one of the strongest predictors for premature delivery and then it was incorporated into the prediction model. Other variables such as preeclampsia and GDM were previously included in a risk-calculating nomogram and machine learning algorithm (10, 34). Hypertensive disorder was one of the known risk factors based on machine learning. Other risk factors included twin pregnancy, systemic lupus erythematosus and short cervical length (35). One of the benefits of machine learning model is the potential to identify risk for idiopathic or spontaneous premature delivery. And it can also consider a wide range of health conditions to infer patterns related to premature delivery (35). Han et al. found that thyroid autoantibodies in the first trimester were associated with an increased risk for hypertensive disorders of pregnancy, and theses associations were independent of thyroid dysfunction (36). In type 1 diabetes, the prevalence of TAI is higher than in healthy population (37). A meta-analysis showed that there was a significant but not strong association between thyroid antibodies and the risk of GDM (38). As indicated above, the pregnant women with TAI are at higher risk of developing preeclampsia and GDM.

To our knowledge, it was the first study that TPOAb positivity and TSH were enrolled in the prediction model of premature delivery. The final model included maternal age, history of premature birth, TPOAb and TSH concentrations in the first trimester, preeclampsia, and GDM. Using these findings, the nomogram was established. The nomogram prediction was supported by the C-index, Hosmer-Lemeshow test, calibration curve and decision curve analysis. The decision curve showed that using a threshold between 5% and 40% to identify pregnant women related with premature delivery would obtain a positive net benefit. The calibration curves of three cohorts were shown to be in good agreement. The strong predictive effect of these parameters was thought biologically plausible and clinically meaningful. Previous preterm delivery and thyroid functions are the most effective predictive factors in early pregnancy, providing important information to clinicians to assist in their decision-making. Preeclampsia and GDM, the main factors influencing pregnancy outcomes, were important variables for preterm birth prediction in this study. Our nomogram is a prediction model that combines preconception and antepartum factors. The dynamic changes in the variables should be noted. The total score of the nomogram will change when preeclampsia is diagnosed after week 20 of gestation or GDM after week 24.

### Strengths and Limitations

Our study had some limitations. What needs to be emphasized is that observational research cannot prove causation, only association. There is currently no single test for predicting premature delivery. Tests such as cervical length and factors related to infection were not included in our model, even though they were thought to be important for preterm birth prediction. Most nulliparous women in our study were considered as being at low risk, dispensing with routine cervical length screening. The sample size of the test cohort was smaller than that of the training and validation cohorts. Study on a large sample size is needed to confirm the effectiveness of a biomarker. We will expand the cohort and strive to validate the accuracy of our predictive tool in the future study.

This study had several strengths. First, we investigated pregnancy-specific cutoff values for TPOAb and TSH concentrations during early pregnancy association with premature delivery. To our knowledge, few studies were conducted to identify the pregnancy-specific cutoffs of TPOAb and the risk of premature delivery. Second, we have developed a nomogram that included new risk variables, such as thyroid function, that had not been used in a nomogram associated with premature delivery before. Third, our study was a large cohort and all variables in the study were based on available data from clinical obstetric history to facilitate the evaluation of pregnant women.

## Conclusion

This study identified the regional and pregnancy-specific cutoff values for TPOAb positivity and TSH concentration associated with premature delivery in singleton pregnant women without pre-pregnancy complications. We have developed a nomogram to predict premature delivery based on thyroid function and other risk factors. The risk calculation with this model was simple, could be used as a clinical decision-making tool.

## Data Availability Statement

The raw data supporting the conclusions of this article will be made available by the authors, without undue reservation.

## Ethics Statement

The study was approved by the Ethics Committee of the International Peace Maternity and Child Health Hospital, School of Medicine, Shanghai Jiaotong University (No. GKLW2019-16). The data analysis procedures followed the guidelines in the Declaration of Helsinki.

## Author Contributions

All the authors contributed to the work. This study was designed by JF, YM, and JL. YM and JL performed the statistical analysis and wrote the manuscript. JF reviewed and edited the manuscript. All authors read and approved the final version of this manuscript.

## Funding

This work was supported by the Medicine and Engineering Interdisciplinary Research Fund Shanghai of Jiao Tong University, China (grant number ZH2018QNA34) and the National Key Research and Development Program of China (grant number 2019YFA08026604). This work was also supported by grants from the National Key Research and Development Program of China (grant number 2018YFC1004602). The funders had no role in the study design, data collection, data analysis, data interpretation, or writing of the manuscript.

## Conflict of Interest

The authors declare that the research was conducted in the absence of any commercial or financial relationships that could be construed as a potential conflict of interest.

## Publisher’s Note

All claims expressed in this article are solely those of the authors and do not necessarily represent those of their affiliated organizations, or those of the publisher, the editors and the reviewers. Any product that may be evaluated in this article, or claim that may be made by its manufacturer, is not guaranteed or endorsed by the publisher.
